# Can humans perceive the metabolic benefit provided by augmentative exoskeletons?

**DOI:** 10.1186/s12984-022-01002-w

**Published:** 2022-02-26

**Authors:** Roberto Leo Medrano, Gray Cortright Thomas, Elliott J. Rouse

**Affiliations:** 1 Department of Mechanical Engineering, University of Michigan, Ann Arbor, 48109 USA; 2 Department of Electrical Engineering and Computer Science, University of Michigan, Ann Arbor, 48109 USA; 3 Robotics Institute, University of Michigan, 48109 Ann Arbor, USA

**Keywords:** Exoskeleton, Metabolic rate, Perception, Psychophysics, Biomechanics

## Abstract

****Background**:**

The purpose of augmentative exoskeletons is to help people exceed the limitations of their human bodies, but this cannot be realized unless people choose to use these exciting technologies. Although human walking efficiency has been highly optimized over generations, exoskeletons have been able to consistently improve this efficiency by 10–15%. However, despite these measurable improvements, exoskeletons today remain confined to the laboratory. To achieve widespread adoption, exoskeletons must not only exceed the efficiency of human walking, but also provide a perceivable benefit to their wearers.

****Methods**:**

In this study, we quantify the perceptual threshold of the metabolic efficiency benefit provided during exoskeleton-assisted locomotion. Ten participants wore bilateral ankle exoskeletons during continuous walking. The assistance provided by the exoskeletons was varied in 2 min intervals while participants provided feedback on their metabolic rate. These data were aggregated and used to estimate the perceptual threshold.

****Results**:**

Participants were able to detect a change in their metabolic rate of 22.7% (SD: 17.0%) with 75% accuracy. This indicates that in the short term and on average, wearers cannot yet reliably perceive the metabolic benefits of today’s augmentative exoskeletons.

****Conclusions**:**

If wearers cannot perceive the benefits provided by these technologies, it will negatively affect their impact, including long-term adoption and product viability. Future exoskeleton researchers and designers can use these methods and results to inform the development of exoskeletons that reach their potential.

## Background

The purpose of augmentative exoskeletons is to help people exceed the limitations of their human bodies. These technologies apply mechanical assistance to the joints of the legs during locomotion, thereby reducing the physical demands on the wearer’s neuromuscular system. The potential uses for these technologies are broad and impactful, including assisting people’s abilities to walk, run, jump, and/or carry loads. Consequently, lower-limb exoskeletons may improve the mobility of people with disabilities, as well as those completing sustained, physically demanding activities (e.g. first responders, postal/supply chain workers, and military personnel, among others). Recently developed systems for human augmentation applications are untethered [1–3], lightweight [1, 4, 5], and powerful [6, 7]. While recent work has been encouraging, an ongoing challenge has been quantifying the success of these systems; the quantification of an exoskeleton’s ability to reduce the metabolic expenditure of walking (i.e. calories burned) has emerged as a focus of the field [8].

Exoskeleton researchers have focused on the reduction of metabolic rate because it is intuitive, measurable, and supported by previous research. State-of-the-art exoskeletons have consistently reduced the metabolic expenditure needed for walking by approximately 14% relative to not wearing an exoskeleton [2, 5, 9–14]. These exoskeletons apply powered assistance at either the ankle joint [5, 9–14] or hip joint [2] and implement control strategies that operate in tandem with the wearer to reduce their metabolic expenditure. Intuitively, if an exoskeleton is successful, the muscular effort required will be reduced, which should be reflected in an upstream reduction in the metabolic power required from the wearer. In addition, metabolic expenditure can be objectively measured in a laboratory setting, meaning it does not have the challenge of quantification that plagues other potentially subjective metrics of success (e.g. comfort, stability, or preference, among others).

There is mounting evidence that humans may be able to ‘subconsciously’ perceive their metabolic rate, but it is not yet known whether these changes can be perceived consciously. Donelan *et al*. showed that people choose step widths that minimize their metabolic rate during walking [15]. Subsequently, Selinger et al. demonstrated that exoskeleton wearers can re-optimize their gait patterns to minimize their metabolic rate when manipulated externally (with an exoskeleton) [16]. That is, the resistance of a knee exoskeleton was varied to incur a metabolic penalty during normative walking patterns, and participants needed to modify their gait patterns to reduce the superimposed metabolic burden. Participants converged to non-normative gait patterns that minimized metabolic rate, but this optimization did not occur spontaneously outside the laboratory [17]. Since exoskeleton wearers choose gait patterns that reduce metabolic rate, we believe this indicates people have some ability to sense this quantity (or something correlated). However, since people do not spontaneously stay or return to their lowest rate, it suggests that exoskeleton wearers do not have conscious knowledge of their metabolic rate or its gradient.

Conscious perception is a critical part of decision making. For an exoskeleton to appear valuable to its potential wearer, it must provide an experience that illustrates this value. Furthermore, this value must offset the potential “costs” of exoskeleton use. For example, without an intuitive and perceivable understanding of value, potential users may be unlikely to adopt exoskeletons with known disadvantages (e.g. monetary cost, discomfort, or being unfashionable). Previous research in the field of management science has investigated the implications of perceived value in technology adoption; one relevant framework proposed by Davis is the Technology Acceptance Model (TAM) [18]. In this model, Davis found a significant correlation between the consciously perceived usefulness of software and users’ intent to adopt the software [18]. More recently, King and He found that this relation was generalizable across many different technologies [19], such as broadband internet [20], telemedicine [21], and smart watches [22]. Thus, when potential exoskeleton users, manufacturers, and others are weighing the choice to adopt or purchase an exoskeleton, the consciously perceived value must outweigh the price, weight, aesthetics, and other costs of wearing a lower-limb exoskeleton.

The field of psychophysics focuses on quantifying human perception broadly [23]; for example, sensing may involve perception of images, temperatures, sounds [24], or metabolic rate. Forced comparison between two stimuli (such as asking which of two lines appears longer) across different trials is a powerful method for determining how humans can perceive changes in stimuli. This pair of stimuli is composed of a reference, which usually remains constant across trials, and a comparison, which changes from trial to trial. By analyzing large numbers of these comparisons, a perceptual model can be built that encodes and quantifies people’s perceptual performance. The input to this model is the true difference between the reference and the comparison, and the output is a probability of the comparison being perceived as different from the reference. The model predicts that stimuli are accurately perceived when the difference between the stimuli is large, but that human perception becomes essentially random when the difference is small. These models are often visualized using a psychophysical curve [25], typically a sigmoid function. In general, a single psychophysical curve pertains to the specific reference stimuli about which the test is conducted.

The steepness of a psychophysical curve quantifies perceptual ability; namely, the smallest difference in stimuli that can be perceived reliably. Using a threshold for reliability of 75% [26], this delta is known as the Just Noticeable Difference (JND). The JND has been used to quantify meaningful differences in visual acuity [27], sound [28], taste [29], and weight [30]. Recently, wearable robotics researchers have begun to quantify the JND of various factors in the design and control of wearable robotic systems, including perception of prosthetic ankle stiffness by users [31] and clinicians [32], environment stiffness [33] and viscosity [34], electrical stimulation of the residual limb [35], and vibrations of an osseointegrated prosthesis [36].

In this study, we characterized exoskeleton users’ conscious perception of their metabolic rate during assisted walking by quantifying the JND of metabolic rate changes. Understanding the human perceptual ability to sense this change is important because it has emerged as the gold standard by which exoskeletons are designed, controlled, and assessed. If exoskeletons are developed to impact a metric that is not perceivable by the user, it will likely hinder widespread success. To this end, we indirectly imposed different metabolic rates sequentially during walking by adjusting the assistance provided from bilateral ankle exoskeletons. Simultaneously, we recorded whether users perceived their metabolic rate to have increased or decreased as the control strategy changed. We aggregated these data to estimate the JND for changes in metabolic rate. The contribution of this work includes new fundamental knowledge of how metabolic rate can be sensed during locomotion and a new benchmark for future exoskeleton developers who desire perceivable impact on metabolic expenditure. In addition, these results underscore the need for new metrics of exoskeleton success that are aligned with the value and experience of the user.

## Methods

### Participants

In this study, ten able-bodied participants (N = 10, 2 female, 8 male; age = 22.5 ± 3.17 years; mass = 70.9 ± 11.9 kg, Table. 1) walked using bilateral ankle exoskeletons on a treadmill. The required number of participants was chosen based on a power analysis to quantify a JND of 15% with 80% power and 5% type 1 error rate. We chose 15% as this was representative of the reductions achieved by the best performing lower-limb exoskeletons [37–39]. All participants provided written informed consent before participation. The study protocol was approved and overseen by the Institutional Review Board of the University of Michigan Medical School.

**Table 1:** Participant data

Subject	Responses	Gender	Weight (kg)	Age (years)
1	53	M	52.2	20
2	100	M	74.0	23
3	34	F	72.0	21
4	100	M	86.0	24
5	100	M	78.5	21
6	100	M	74.0	23
7	100	M	82.5	24
8	100	M	59.0	19
9	100	F	53.5	20
10	100	M	77.0	30

### Experimental protocol

#### Walking protocol

Participants experienced numerous metabolic rate changes in sequence that stemmed from the assistance provided by the ankle exoskeletons. Participants walked for 20 minute blocks, where each block consisted of 10 trials in series. Following each pair of trials, participants responded regarding which condition they perceived had a higher metabolic rate by agreeing or disagreeing to the binary question “is the current level of exertion higher than the previous level of exertion?”. Participants responded non-verbally with either a ‘thumbs up’ or a ‘thumbs down.’ Thus, each block consisted of nine comparisons across ten trials and participants completed approximately 11 blocks across three to four days of data collection. We chose the two minute walking duration for each trial to balance metabolic estimation quality with experiment duration; Zhang et al. demonstrated that the metabolic estimation error with two minutes of data is approximately 2$\%$ [40]. The two-minute trial duration also allowed the participants adequate time to experience and react to each walking condition.

Prior to the experiment, participants familiarized themselves with several aspects of the experimental protocol. Participants read a lay explanation of metabolic rate to familiarize themselves with the concept. Next, participants were primed to react to their feeling of general exertion by reading the instructions of the Borg Rating of Perceived Exertion [41, 42]. We chose to have the participants read information on the Borg Scale because it has previously been demonstrated to produce accurate estimates of exertion [43]. Finally, participants underwent a four-minute acclimatization period in which they were exposed to different representative exoskeleton behaviors that spanned what could be encountered during the experiment.

**Fig. 1:**
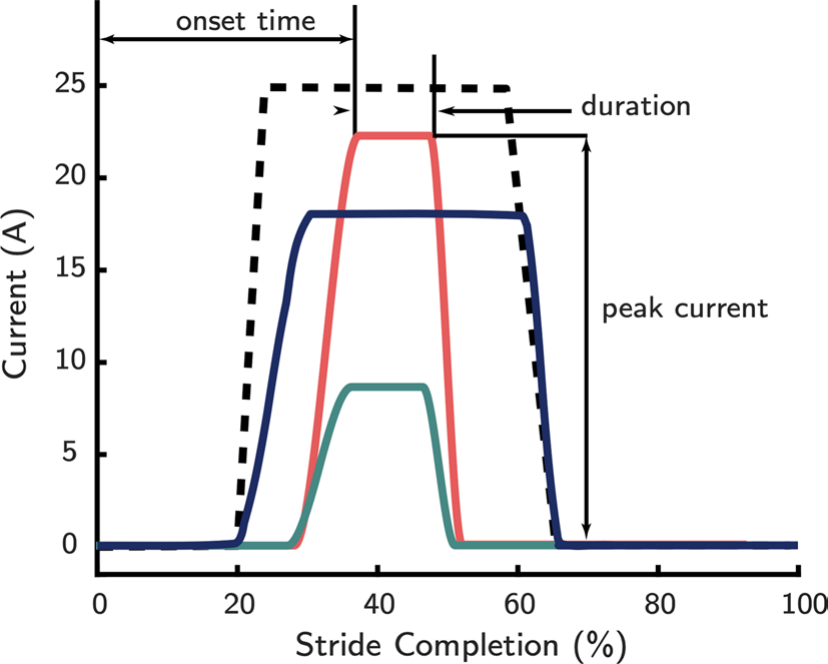
Sample exoskeleton current profiles used in this experiment (colored lines). The profiles resembled square pulses and were parametrized using the following parameters: the peak current of the profile, the onset time of the profile, and the duration of the profile. The total bounds of possible current profiles are delineated by the black dashed line. The profiles’ currents were mapped to motor torques through the motor torque constant and exoskeleton’s transmission

#### Exoskeleton control

**Fig. 2:**
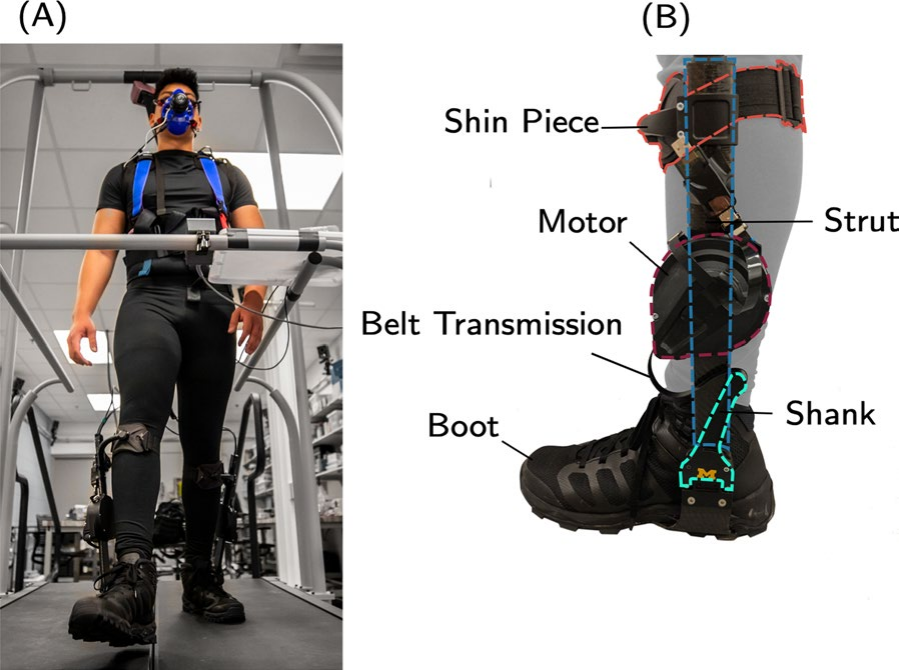
**A** The exoskeleton-human system (picture taken prior to the COVID-19 pandemic). Participants walked on a treadmill and experienced different changes to their metabolic rates, which were measured using indirect calorimetry. **B** The Dephy ExoBoot ankle exoskeleton used in the physical experiment. A brushless DC motor mounted on a rigid shank assists the user by generating torque through a belt drive transmission that applies force on a boot-mounted strut. The exoskeleton is securely attached to the user via a shank attachment that transmits the actuator’s torque to the leg

We used bilateral ankle exoskeletons (Dephy ExoBoot, Dephy Inc. Maynard MA) to manipulate the metabolic rate of the wearer. The exoskeleton (Fig. 2B) used electric motors ($\sim$300 W) and flat cable transmission ($\sim$15:1) to apply plantarflexion assistance during walking. The assistance was governed by parameterized current profiles that resembled a square pulse (see Fig. 1). The current profiles were governed by three parameters; we manipulated the onset timing, pulse magnitude, and pulse duration. We chose (i) onset timings from a uniform distribution bounded between 25% and 50% of stride time, (ii) pulse magnitudes from a uniform distribution bounded between 15 A and 25 A (corresponding to approximately 12 and 20 Nm with the ExoBoot’s nonlinear transmission), and (iii) pulse durations from a uniform distribution with variable bounds. The variable bounds for the pulse duration depended on the sample drawn from the onset timing distribution such that the square pulse had a minimum duration of 10% of stride, and a maximum duration of 60% of stride time. Onset timings that occurred earlier than 30% of stride were additionally constrained to have a minimum pulse duration of 20% of stride, which was imposed to prevent excessive device wear. We chose these bounds as they have been shown to significantly alter participant metabolic rate, and thus allow us to sample as wide an energetic range as possible [12, 44, 45] while balancing device integrity and user safety. The current profiles were described using the stride completion percentage to mitigate any variations in step length or cadence that occurred during the trial. Thus, we inferred the stride completion percentage using heel-strike events. We detected these events by thresholding the onboard accelerometers (MPU-9250, Invensense, San Jose, CA) [46, 47].

#### Metabolic rate sensing

Participants walked with a randomized torque profile for 2 min, which produced a first-order dynamic response in metabolic rate [48]. We measured participant metabolic rates through indirect calorimetry [49] (COSMED K5, Rome IT) (Fig. 2A). We estimated the user’s steady state metabolic rate by fitting a first-order response [44, 48] to the breath-by-breath transient data gathered over the two minutes for each trial, with the steady state value representing the trial’s metabolic rate. Prior to undergoing the walking protocol, participants stood still for four minutes to obtain their baseline metabolic rates. Each participant’s standing metabolic rate was computed as the average rate over this four-minute interval. The standing metabolic rate was subtracted from each trial’s metabolic rate measurement to isolate the metabolic effects of exoskeleton assisted locomotion (i.e. net metabolic rate).

### Psychophysical function fitting

To estimate the JND of metabolic rate, which denotes the magnitude of change necessary for consistent perception, our experimental protocol requires the normalization of metabolic rates. That is, to compare across sequential trials with differing references, normalization is needed to combine these data to obtain a single JND for each subject under the assumption of a constant Weber Fraction (see Limitations subsection) [50]. The Weber Fraction (WF) [51] is a metric that captures the differences in perceptual thresholds that are dependent on the magnitude of the reference stimulus used in the comparisons. By definition, the WF is the JND divided by the reference stimulus, thus it represents the percent change from the reference stimulus that is perceivable. For a wide range of stimulus magnitudes, the WF can be modeled by a constant [52].

For normalization, consider a sequential pair of metabolic rates A then B; we normalized B (the comparison) as a percent change in rate from A. Note that rate A is the reference that changes from trial to trial. We used the normalized metabolic rate differences and corresponding participant responses to fit a psychometric function. The psychometric function then provided JNDs with units of percent change of metabolic rate (rather than absolute units (W/kg)). The JND is then equivalent to the WF expressed as a percentage. This is advantageous as it allows the resulting JNDs to characterize the perceptual thresholds of different tasks that may have different baseline rates (in W/kg), such as locomotion tasks at different walking speeds—the minimum perceivable change for those tasks would still be the percentage change relative to any baseline metabolic rate, as long as the comparison pertains to stimuli where the Weber Fraction is constant.

A logistic psychometric function was used to model participant responses. This model predicted the probability that the participant would choose “the comparison is greater” as a function of the normalized metabolic rate difference between the two trials. Using the convention from above, the psychophysical curve predicts the probability that rate B is greater than rate A, as a function of the relative difference between A and B. The logistic function of (reference-normalized) stimulus *x* had the following form,

1 $$\begin{aligned} \Psi (x,\alpha ,\beta ,\gamma ,\lambda ) = \gamma + \frac{1 - \lambda - \gamma }{1 + e^{-\beta (x - \alpha )}} \end{aligned}$$

where $\Psi (x)$ was parametrized by the following variables: the experimental lapsing rate $\lambda$, which was fixed at the commonly used value of 0.02 [23]; the false positive rate $\gamma$, which was fixed at the lapsing rate since participants underwent a stimulus discrimination task [23, 53]; the logistic function’s threshold point $\alpha$ on the x-axis, which anchors the center of the logistic curve and was set to 0; and the parameter $\beta$ which governs the slope of the logistic function and is the only degree of freedom estimated during the fitting procedure.

Using the modeled psychometric curve, we quantified the JND which represents the minimum change in metabolic rate that must occur before an observer can reliably perceive with 75% accuracy [54]. The JND is calculated by taking the difference between the values of *x* at $\Psi (x)=0.75$ and $\Psi (x)=0.25$ and dividing the difference by two. By fixing the other parameters of $\Psi (x)$ at the values specified, the JND thus depended only on $\beta$

2 $$\begin{aligned} \mathrm {JND} = {k}/{\beta }, \end{aligned}$$

with a scale constant *k* in terms of the fixed parameters,

3 $$\begin{aligned} k = \frac{1}{2}\ln \left[ \frac{(0.75 - \gamma )(1 - \lambda - 0.25)}{(1 - \lambda - 0.75)(0.25 - \gamma )}\right] . \end{aligned}$$

Shallower slopes (indicating less sensitivity) caused higher JNDs, while steeper slopes (indicating higher sensitivity) caused lower JNDs.

### Survey on fitness correlates

As a secondary outcome, we investigated potential links between the magnitude of the estimated JNDs and subject-specific fitness variables. At the close of the experiment, subjects completed a survey in which they indicated how many hours per week they typically performed endurance activities (*e.g.* running), how many years they had spent performing these athletic activities, and their Body Mass Index (BMI). The intent of this survey was to provide initial investigation into potential underlying factors that may influence metabolic perception.

## Statistics and comparisons

A separate logistic model was fit for each participant using Bayesian analysis [55]. This approach yielded a posterior distribution of JND estimates for each participant. From this posterior distribution, we extracted the maximum likelihood estimate for each participant, which was considered the estimated JND [56]. Our approach of using Bayesian estimation enables quantification of both the JND value for each subject in addition to the uncertainty about our estimates. We chose Bayesian estimation because preliminary work indicated the conventional Maximum Likelihood Estimation approach could fail to converge [57]. We conducted our Bayesian analysis using the PyMC3 library in Python [58]. Each participant’s prior distribution of JND estimates was chosen as a uniform distribution between 0% and 70%, representing a plausibly large range of perceptual abilities.

The posterior JND distributions were obtained by updating our prior distributions using the participant response data. We used the No-U-Turn Sampler (NUTS) [59] strategy—a Markov Chain Monte Carlo (MCMC) algorithm—to numerically approximate the posterior distribution of possible JND values; we used four sampling chains with 8000 tuning iterations and 4000 posterior predictive samples. We chose these values to balance computation time and accuracy. The JND with the highest likelihood in the posterior distribution was the nominal JND estimate. Each posterior also yielded a 95% credible interval for the JND estimates.

Our approach using Bayesian statistics enabled investigation of several assumptions made about the JND distributions. We compared three different JND models using the Watanabe-Akaike Information Criterion (WAIC) metric, which evaluates the predictive power of models and corrects for the number of model parameters to favor parsimony [60]. The three competing models were: (i) Pooled—assuming all participant JNDs arose from a single posterior distribution, and which therefore does not allow for inter-participant differences (this model thus has a single constant WF); (ii) Independent—assuming each participant has a single independent JND (and a constant WF), which allows for inter-participant differences in JND posterior distributions; and iii) Variable WF—assuming each participant can have two JNDs. The first JND was calculated using the metabolic data that corresponded to absolute reference costs in the lower half of that participant’s reference cost magnitudes, and the second JND was calculated using the metabolic data in the upper half of reference magnitudes. Thus, this model featured a non-constant WF in which the JND varies based on the absolute magnitude of the reference cost.

In each model, our parameter estimates were informed by the data, yielding posterior distributions over all possible parameter estimates. In the pooled model, the single JND estimate predicted the responses of all participants; in the independent model, each participant’s responses were predicted by individual JND distributions which we estimated; and in the variable-WF model, each participant had two different JNDs for references above or below the average reference value. The pooled model featured 2000 tuning iterations and 2000 posterior predictive samples for reduced computational time, given the greater number of responses used as input. The remaining models used our default settings of 8000 tuning samples and 4000 posterior predictive samples due to the relative sparsity of the data and the complexity of the models.

## Results

**Fig. 3:**
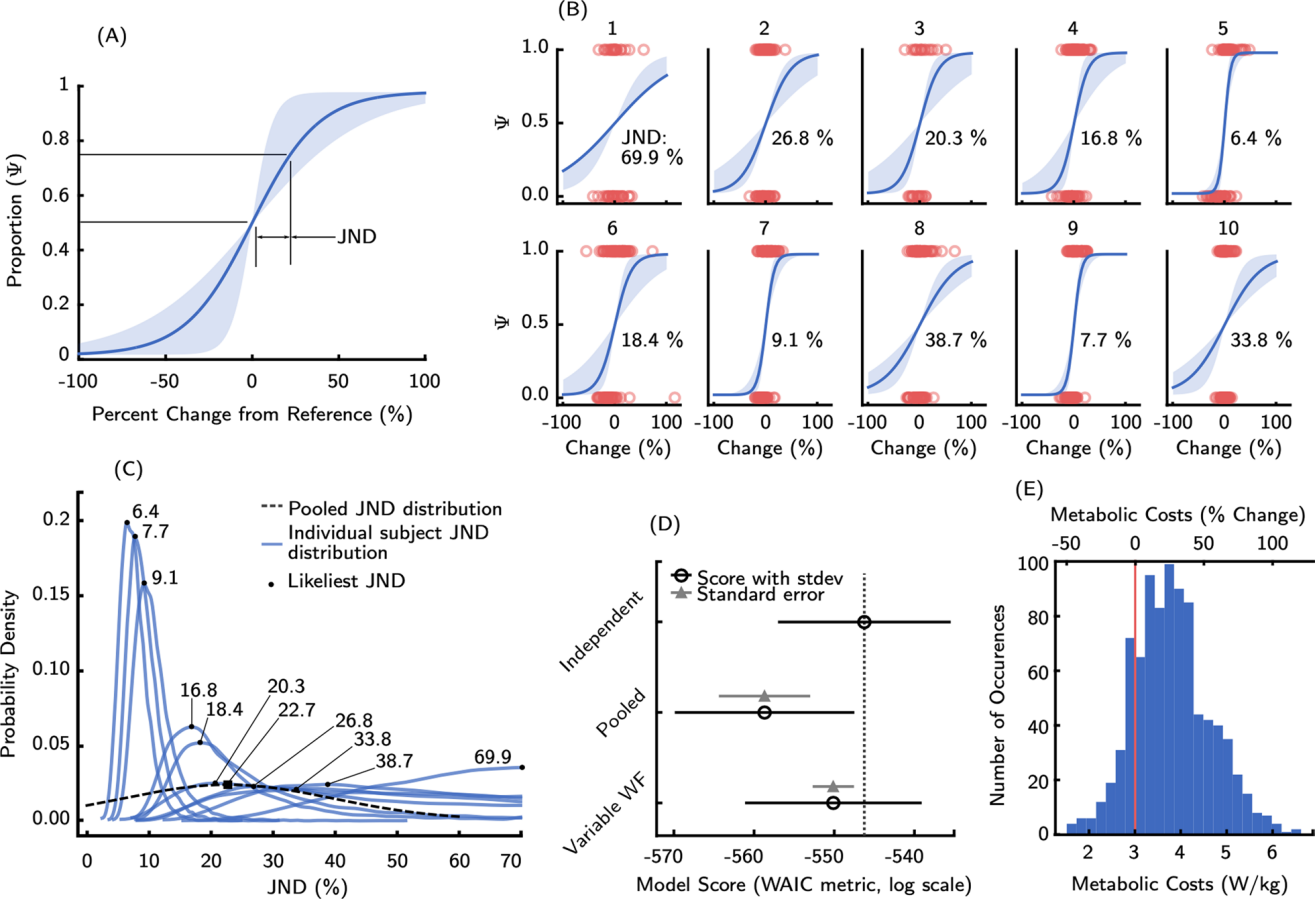
**A** The psychophysical curve corresponding to the inter-participant average (solid blue, 22.7%) with one inter-participant standard deviation (shaded, ± 17.0%). **B** Participant-specific data: the likeliest psychophysical curve for each participant (solid blue), participant responses (red circles), and the 95% credible interval of possible curves from the posterior distribution (shaded blue). **C** The posterior distribution for the inter-participant psychophysical curve (dashed black) vs. the posterior distributions for each participant with modeled inter-participant JND differences (blue). The inter-participant model posterior distributions show clear differences between participants and thus proved a better choice of model. **D** A comparison of different JND models using the Watanabe-Akaike Information Criterion (WAIC) metric. A higher WAIC score (black circle, standard deviations given by black lines) indicates a better model. The best model has a light gray dotted line through its empty circle to aid in comparison. Grey triangles indicate the difference in WAIC between that model and the top model (standard error given by grey bars). **E** The absolute range of reference costs aggregated across all participants. The vertical red line denotes the average net cost of walking at 1.25 m/s across different studies [61]

The average inter-participant JND was 22.7% (standard deviation (SD): 17.0%) (Fig. 3A). Many participants were highly attuned to the changes in their metabolic energetics, while others were less perceptive, as evidenced by the high standard deviation of the estimates (Fig. 3B). We used a one-sample Kolmogorov-Smirnov test in matlab to verify that the independent JND estimates from the participants were normally distributed. The lowest estimated JND was 6.4%, while the highest was 69.9%. The standard error of the mean (SE) was 5.35%. Our confidence in each participant’s JND estimate was given by their respective JND posterior distributions, which represent the distribution over potential JNDs of each participant (Fig. 3C). Differences in the JNDs can then be observed by comparing the shapes of these distributions; for example, a narrow distribution with a defined peak at a low value represents a participant who is highly attuned to changes in energetics, while a flattened distribution with a peak at a high value denotes a participant with a greater JND and less sensitivity.

We used the WAIC metric [60] to identify the psychophysical model that best describes our data (Fig. 3D). This metric evaluates the predictive power of each psychometric model [62] and corrects for the number of parameters to favor parsimony. The best model was the independent-JND model with a constant WF (described in (ii) above in Statistics and Comparisons), which obtains the highest WAIC score and is outside the standard error regions of both competing models.

**Fig. 4:**
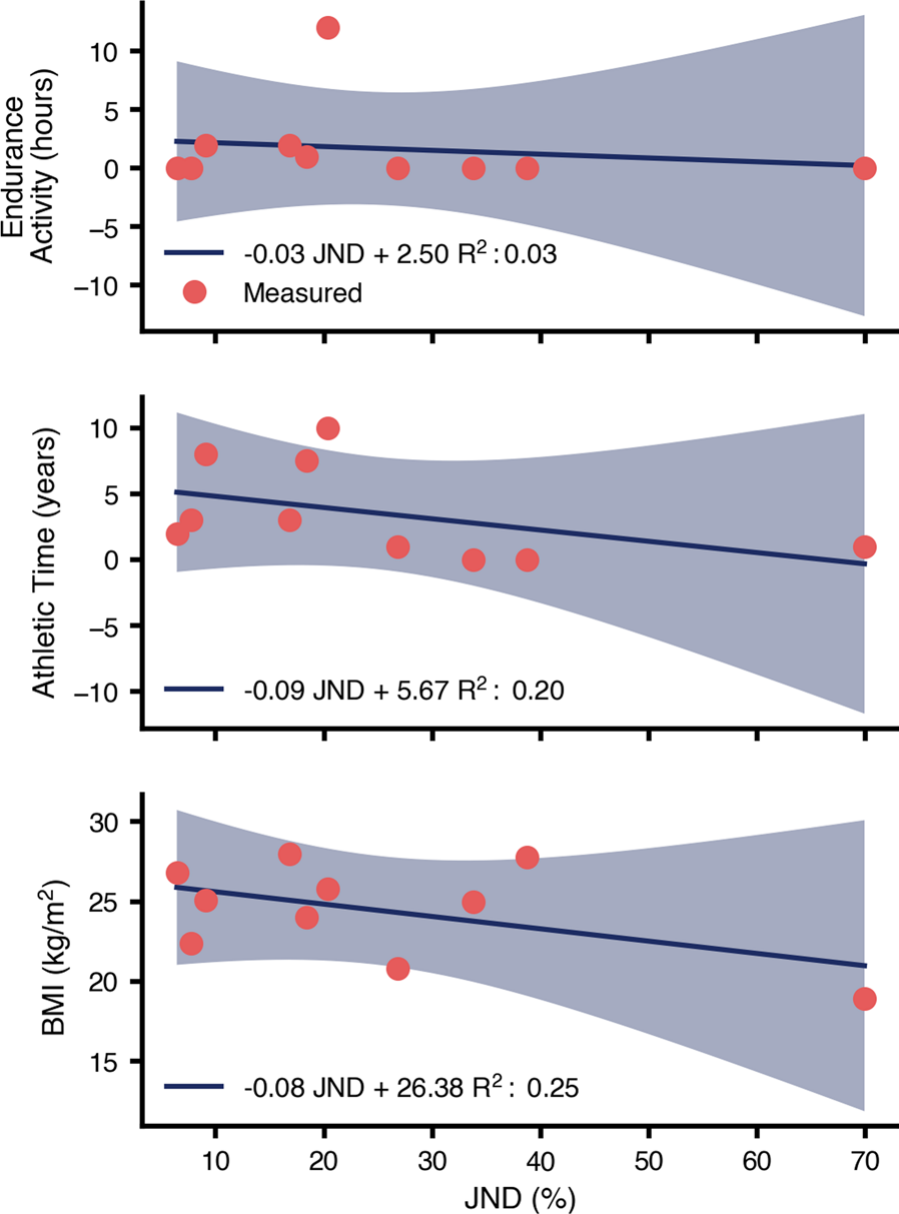
Fitness variables shown across estimated JNDs: Hours spend performing endurance activity (e.g. running exercise), years spent performing athletic activity, and Body Mass Index (BMI). The regressed best-fit lines are shown in blue, with individual responses in red. The 95% confidence intervals are shown by the shaded region. No relationships were statistically significant

We found no significant relationships in our exploratory analysis of fitness correlates. We regressed lines of best fit to identify any relations between the magnitude of the independent JNDs and the fitness variables (Fig. 4). We conducted F-tests to detect significance of the slope ($\alpha = 0.05$). No significant relationship was found between the fitness variables and the magnitude of the estimated JNDs.

We examined the range of absolute metabolic rates experienced by participants in our protocol and verified that the metabolic rates humans experience while walking with an assistive exoskeleton were included in this range ($\sim$10% reductions from unassisted walking, see Fig. 3E). As the WF is modeled as locally constant over this range, the JNDs estimated in this work pertain to the perception of energetics in both augmentative and resistive assistance regimes.

## Discussion

**Fig. 5:**
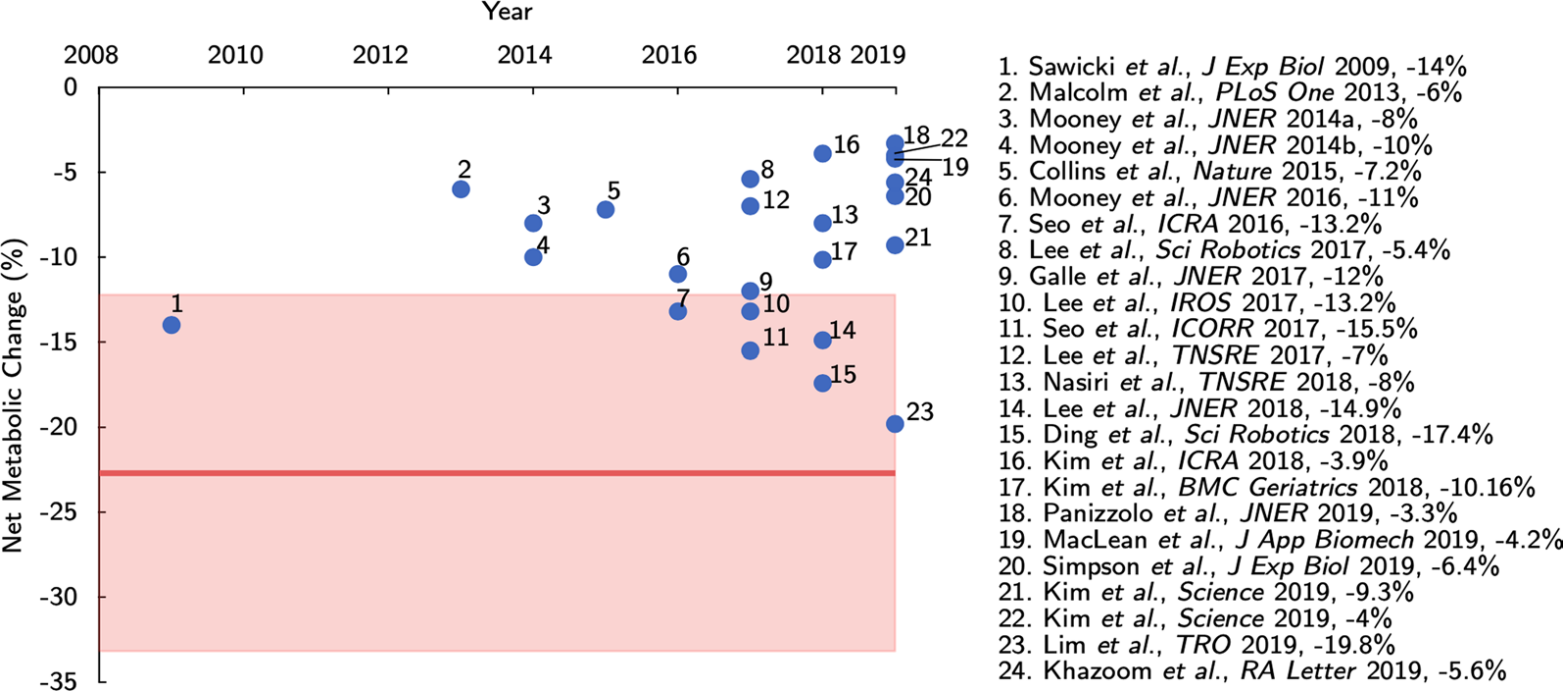
The average JND magnitude (solid red) plotted against the state-of-the-art in exoskeleton-driven metabolic rate reductions. The 95% confidence interval for the standard error of the mean of 5.35% is shown (shaded red). As of the date of writing, no published device (to the authors’ knowledge) exists that would cause a perceivable short-term energetic benefit to the average wearer. Data reproduced with permission from [8]

On average, modern augmentative exoskeletons do not yet provide a metabolic benefit sufficient to exceed the perceptual threshold of human energetics. We demonstrated that the average Just Noticeable Difference (JND) of metabolic rate was $22.7\% \pm {5.35}\%$ (SE). This is substantially greater than the typical reductions obtained using state-of-the-art exoskeletons over the past decade (see Fig. 5) [8]. While some studies have shown metabolic reductions greater than 15% [37–39], most research has demonstrated more modest reductions. The mean reduction in metabolic rate over the past decade is $\sim$9.6% ± 4.5% (SD) (averaged from studies in Fig. 5). Based on the inter-participant psychophysical curve obtained in this work, there is a 61% likelihood an average user would perceive a 9.6% change in metabolic rate, when compared to walking without an exoskeleton (50% accuracy would be a random guess). Thus, based on the metabolic rate reductions provided to date [8], the typical benefit from these devices is unlikely to yet be a critical factor in the short-term, conscious perception of exoskeleton use. The perceptual threshold presented in this work (i.e. the JND) can act as a useful benchmark for future exoskeletons designed to noticeably improve walking energetic efficiency.

For augmentative exoskeletons to demonstrate value to their wearers, the benefits provided should be perceivable in the short term. Given that the state-of-the-art exoskeletons do not yet provide a metabolic benefit that can be sensed reliably (i.e. metabolic benefit $>22.7\%$), this reduction in metabolic rate is not likely to be a driving factor in short-term decision making. Given the short-term nature of the trials in this study, it is possible the reduction in metabolic rate is more perceivable over an extended period of use. While this could positively impact user experience, perception over a longer duration may also lead to challenges in experience and adoption. Prior work in economics has demonstrated that a benefit provided in the future is less valuable when compared to a more immediate benefit (i.e. temporal discounting) [63–69]. Thus, we believe exoskeletons will be most successful if the metrics used to develop these technologies are aligned with what is perceivable and valuable to the user in the short term. Understanding if and how longer-term energetic reductions are perceivable, in addition to the impact of temporal discounting, are important avenues of future study.

Metabolic rate reduction is currently the “gold standard” for augmentative exoskeletons, which is supported by its role in the reduction of joint mechanical power, previous biomechanical studies, and its objective measurability. However, our results demonstrate that the current reductions in metabolic rate are not yet broadly perceptible in the short term. The difficulty of perceiving changes to metabolic rate motivates the consideration of alternative metrics which may be more clearly perceivable by users, including reduction of muscle fatigue [70–72], peak joint forces in arthritic joints [71, 73, 74], and user preference [31, 75–77]. The development of perceivable and meaningful metrics to quantify success in future exoskeletons is an important challenge for the field.

Previous work investigating the perception of exertion has shown lower thresholds for exertion during exercise cycling [78]. Haile et al. applied the method of adjustment [23] to cycling intensity, arriving at a threshold of 0.15 L/min $\dot{VO_2}$, but did not provide a resting metabolic rate for their participants. Using the resting rate estimates provided in [79], this equates to a JND of $\sim$10%. There are several possible explanations for the differences from our results. For example, the method of adjustment can lead to lower JND estimates [80], and is known to be less reliable [81–83] than forced-choice experiments. Additionally, the exertion levels tested were substantially greater than what was tested in our experiment, and thus might have occurred in the perceptual regime where the WF was non-constant. Lastly, their method of moderating exertion used only a single variable (cycling resistance), which is susceptible to confounding factors. That is, cycling resistance will vary proportionally with muscle loading, which can be more easily sensed through the Golgi tendon organs, mechanoreceptors, and other mechanisms. Thus, any perceptual thresholds calculated using these sensations could be confounded to underestimate the true JND of exertion because the participants could intuit a mapping between the easier-to-perceive cycling resistance and the harder-to-perceive metabolic effort.

Researchers have established that humans will seek energetically optimal gaits, even when metabolic rate changes are far below our estimate of the perceptual threshold for metabolic rate (i.e. $\sim$5% rather than 22.7%) [16, 84]. One potential explanation for these differences is that our experiment measures conscious perception of changes in metabolic rate, whereas this prior work has allowed for potential subconscious sensing contributions from sensorimotor system and autonomic nervous system [85, 86]. The literature suggests that humans rely on a combination of different afferent signals, such as heart rate or muscular strain, to generate a gestalt perception of exertion in ways that are not yet fully understood [43, 87–90]. It is also not yet known whether the observed changes in locomotor mechanics that are correlated with metabolic rate are causally linked to those changes.

Participants varied greatly in their ability to perceive changes to their metabolic rate. In this study we investigated whether the JND was more appropriately modeled as a constant value or a person-specific value. Using the Watanabe-Akaike Information Criterion (WAIC) metric—a modern Bayesian tool for comparing the quality of models—we found that the data were better fit by the model where each participant had their own independent JND (see Fig. 3D). While the inter-participant mean JND value was greater than the metabolic benefits provided by modern exoskeletons, our participant pool included three participants who had JNDs below 9.6% and thus would likely perceive benefits from these technologies [8] (see Fig.  3C).

Although our study was not powered to identify significant relationships between fitness and perception, we investigated this potential link by regressing linear models between the estimated JND and relevant fitness variables (hours weekly spent performing endurance activities, years spend performing athletic activities, and BMI). We failed to detect any significant relationships between the fitness variables and the magnitude of the estimated JNDs. Future work is needed to study both the physiological mechanisms that underlie variability in perception of energetics, as well as discover methods to identify those users who may have better perception.

In our experiment, the WF—the ratio of the JND (in absolute units of W/kg) to its corresponding reference—is constant with respect to reference magnitude over the range of references tested. The Variable WF model we evaluated, in which each subject’s JND was dependent on the absolute magnitude of the reference, was inferior to a model in which each subject had a single JND and constant WF. Given the inter-participant range of reference costs experienced (from $\sim$1.5 to 6.6 W/kg, see Fig. 3E), we believe our experiment occurred in the mid-section of the perceptual regime, rather than the extremes around which the WF will rise steeply in many cases [51]. This distribution of reference metabolic rates does skew towards relative increases away from the metabolic rates experienced during baseline unassisted walking, as it is far easier to add a metabolic penalty with exoskeleton use. However, in many psychophysical studies, the psychometric curve is assumed to be symmetric about the origin [25, 53, 91–93]—that is, subject perception does not depend on the sign of the comparison. Due to this symmetry and the success of the constant-WF model, we anticipate that the estimated JND pertains to both metabolic benefits and metabolic penalties. Consequently, our results may not only apply to the benefits from modern exoskeletons, but also extend to applications where metabolic penalties may be more relevant (e.g. athletic or endurance training).

### Limitations

The posterior distributions for those participants with low and high JNDs were differently shaped, reflecting a limit on the maximum metabolic rate changes possible via exoskeleton assistance. The exoskeleton used in this experiment was capable of providing a peak torque of approximately 30 Nm ($\sim$ 10 J per stride), which limited the available metabolic rates that could be experienced. The ability to induce a wide array of metabolic rates is important for sampling the psychometric function. To obtain estimates of these functions that have low uncertainty, they must be sampled across both the constant and transitory regions of the psychometric curve [23]. The quality of the measurements is reflected in the posterior distributions for the JND estimates, with high quality measurements resulting in narrow posterior distributions. For participants with smaller JNDs, the limitation on available metabolic rates enabled the sampling of the majority of the relevant areas of the psychometric curve. This allowed us to exclude both excessively large and small estimates for those participants. In contrast, for participants with high JNDs, the imposed energetics spanned a comparatively narrower region of the psychometric function, which only excluded lower JNDs. The posterior distributions for participants with high JNDs was asymmetric, and thus contained greater uncertainty in the upper bound of the threshold. Consequently, any error would likely bias the true JND to be greater than what was measured in this study.

The indirect nature of manipulating energetics via an exoskeleton increased variability in each participant’s JND distribution. In conventional psychophysical studies, researchers have more deterministic control over the applied stimulus under investigation. While exoskeletons are known to influence energetics indirectly through several controllable [11, 12, 44, 94] aspects of the torque profile, metabolic rate also depends on many uncontrollable factors that appear noise-like [49, 79, 95]. This added noise results in sub-optimal sampling of the psychophysical curve that reduces certainty in the corresponding JND estimates [50]. This uncertainty is reflected in the width of the posterior distributions of each participant.

The uncertainty of our results also stems from an experimental limitation in how many trials are feasible. Conventional best practice in the psychophysics literature would recommend $\sim$300 trials [53] when estimating the underlying psychophysical curve; however, in this study we were able to obtain $\sim$100 trials for each participant. The relatively low number of trials was due to the time necessary to obtain responses. In this protocol, participants experienced different metabolic rates in sequence, each of which requires two minutes to estimate the participant’s metabolic rate. To obtain the necessary data for this experiment, participants walked during three sessions spread across three days, with each session lasting four hours. This is in contrast to many studies of human perception, which can obtain experimental data without the time delay of the human cardiopulminary system ($\tau _r$ = 42 s [96]). Consequently, the uncertainty of our estimates was increased by approximately 60% [50] due to the lower number of samples, which is reflected in the inter-participant distribution of the JNDs.

We found that despite the uncertainty in JND estimates, these estimates were relatively insensitive to assumptions in our approach. We used a uniform prior distribution in our analysis that encompassed available JNDs between 0% and 70%. We investigated the sensitivity of our results to the bounds of this prior distribution (i.e. 0% and 70%). We chose our lower bound to reflect perfect human perception, while the upper bound was informed by the reasonable assumption that a human could consistently detect changes in energetics just under those that result from switching from walking to running (a $\sim 100\%$ change [97]). When the bounds of our uniform prior distribution were changed to [0%, 60%] and [0%, 80%], we found that the average inter-participant JND estimate shifted from 22.7 to 21.9% and 23.2%, respectively. These small shifts in the mean JND estimate indicate that our approach is robust to the exact shape of our prior distributions, and are thus well-informed by our sampled data.

Participants responded to questions about exertion, but we are unable to know what specifically drove their answers. Our study relies on participants honestly reporting perceived exertion and not confounding this report with other perceptions, which could include perceptions of assistive torque and assistance timing, as well as higher-level perceptions of the helpfulness of the actuation profile. Our study was designed to mitigate these confounding factors. Participants read a predefined script to help elucidate the concepts of metabolic rate and exertion. The prompt was designed using vocabulary consistent with the Borg Scale, used to assess exertion [41, 43, 89]. Additionally, the torque profile was designed to be intentionally complex (see Methods). That is, the participant’s metabolic rate was induced by the complex interaction of three controller parameters, obscuring any foreseeable relationship with metabolic rate (i.e. “it feels more powerful when it is stronger, which must lower my exertion”). However, if participant’s JNDs were affected by additional informative sources, this would also bias the true JND of metabolic perception to be greater than what was estimated.

## Conclusion

Motivated by the need to develop augmentative exoskeletons that can realize their potential to impact society, we quantified the human ability to perceive the metabolic impact of these technologies. Participants were able to perceive an average change of 22.7% ± 5.35% (SE) in their metabolic rate with 75% accuracy. in short-term testing. Thus, the average user cannot yet consciously perceive the metabolic benefits from the typical modern exoskeleton, which may hinder translation and adoption of these technologies. Our results provide a new benchmark for augmentative exoskeletons that will enable perceivable value to their users. The relatively insensitive perception of metabolic rate also suggests that alternative metrics for exoskeleton success, such as reduced muscle fatigue, loading, or user preference, may be more significant to user experience and exoskeleton success.

## Data Availability

The datasets generated and/or analysed during the current study are available in the CodeOcean repository, https://codeocean.com/capsule/8525772/tree [98].
